# Clindamycin Phosphate-Mediated Enantioseparation of Amino Acids by Capillary Electrophoresis: Molecular Modeling and NMR Insights into the Chiral Recognition Mechanism

**DOI:** 10.3390/molecules31203580

**Published:** 2026-10-09

**Authors:** Mohammed A. A. Khalid, Abdalla A. Elbashir, Munirah A. Alfarhan, Shazalia Mahmoud Ahmed Ali

**Affiliations:** 1 Department of Chemistry, Turabah University College, Taif University, Taif 21944, Saudi Arabia; m.khalid@tu.edu.sa; 2 Department of Chemistry, College of Science, King Faisal University, Al-Ahsa 31982, Saudi Arabia; 3 Department of Chemistry, Faculty of Science Al-Zulfi, Majmaah University, Al-Zulfi 11952, Saudi Arabia

**Keywords:** clindamycin, capillary electrophoresis, amino acids, recognition mechanism, molecular modelling, NMR

## Abstract

In this research paper, chiral separation of three amino acids (AAs), namely, tryptophan (Trp), tyrosine (Tyr), and phenylalanine (Phe), was achieved by capillary electrophoresis with clindamycin phosphate as the chiral selector. Factors affecting the chiral separation, such as the BGE pH, concentration of clindamycin, organic modifier concentration, capillary temperature and applied voltage, were optimized and discussed. A BGE consisting of 80 mM clindamycin phosphate and 40 mM borate at pH 7.6 with 30% methanol was found suitable for chiral separation of tryptophan and tyrosine while the same BGE at pH 7.3 was suitable for enantioseparation of phenylalanine. The optimum capillary temperature and applied voltage were 20 °C and 19 kV. Molecular docking (Glide SP/MM-GBSA) and ^1^H NMR chemical-shift perturbation (CSP) analysis were combined to clarify the chiral recognition of tryptophan, phenylalanine, and tyrosine by clindamycin phosphate. Docking results support clear stereochemical discrimination for Trp, weaker discrimination for Phe, and near-degenerate discrimination for Tyr, and revealed that electrostatic attraction discrimination was the main binding anchor, supported by hydrophobic contacts and hydrogen bonding. The NMR results revealed chemical-shift perturbations and line-broadening patterns consistent with changes in the local chemical environments of clindamycin phosphate regions implicated by the docking models. Pronounced signal broadening in DL-Tyr further revealed dynamic binding behavior that could not be detected by docking alone.

## 1. Introduction

**Figure 1:**
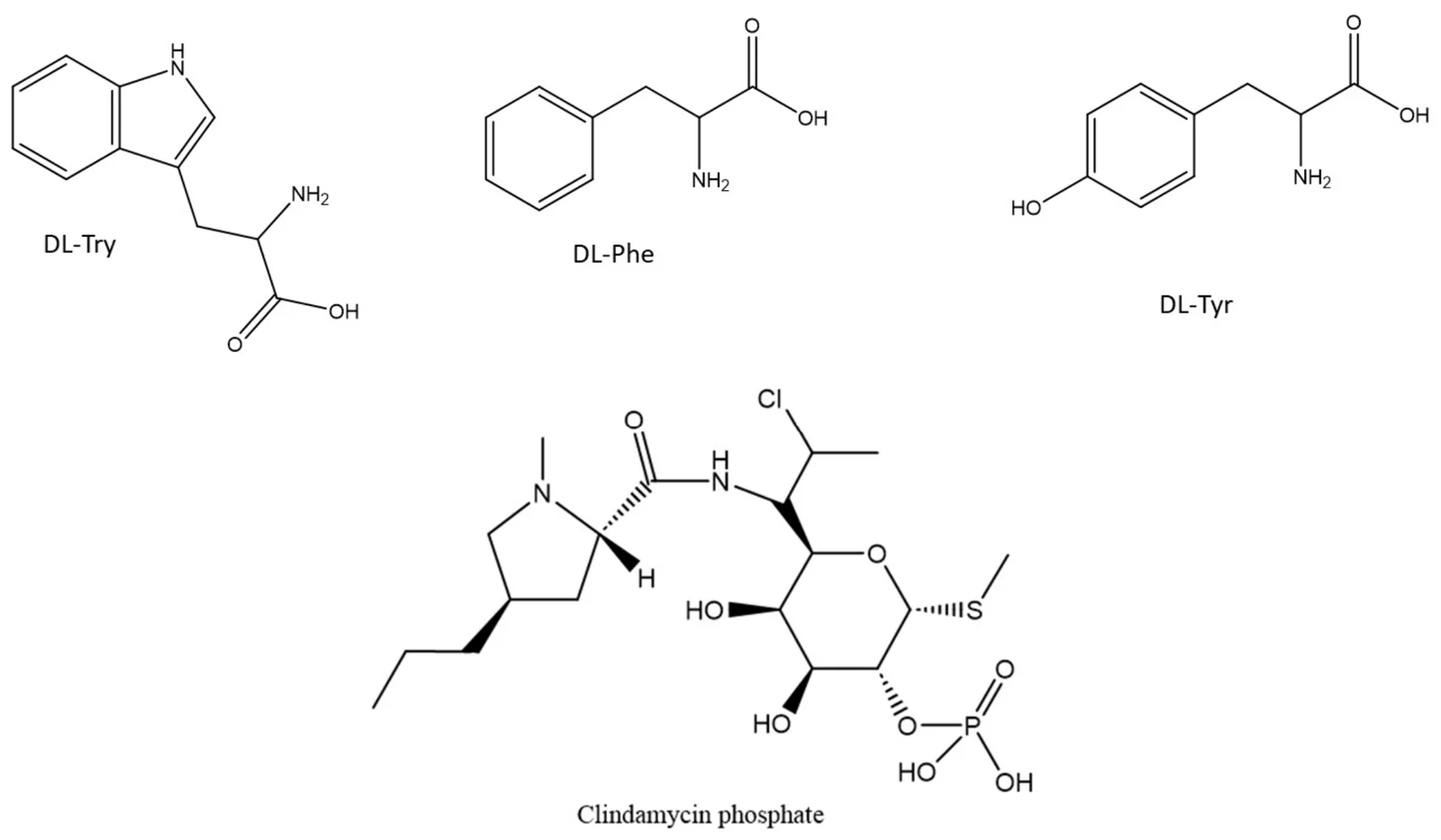
Chemical structures of the L- and D-enantiomers of tryptophan (L-Trp and D-Trp), phenylalanine (L-Phe and D-Phe), and tyrosine (L-Tyr and D-Tyr), together with the chemical structure of clindamycin phosphate (CP).

Aromatic amino acids (AAs), including tryptophan (Trp), phenylalanine (Phe), and tyrosine (Tyr), as shown in Figure 1, are important precursors in the biosynthesis of proteins and numerous phenolic compounds [1,2]. Enantiomerically pure AAs also have broad applications across the food, agrochemical, and pharmaceutical industries. Although L-amino acids are the principal building blocks of proteins, their D-enantiomers have gained increasing interest because of their use in chemotherapeutic agents, antibiotics, fluorescent DNA markers, immunosuppressants, deodorants, sweeteners, pesticides, and other specialized products [2,3]. The contrasting roles of amino acid enantiomers reflect the pronounced stereochemical selectivity of biological systems and highlight the importance of accurately distinguishing and utilizing each enantiomer in diverse scientific and industrial applications [3,4].

Capillary electrophoresis (CE) is the one of the most analytical techniques used in the separation of enantiomers [5,6,7,8,9]. Chiral separation in CE is achieved by adding chiral selectors to a background electrolyte (BGE). A number of chiral selectors have been used such as cyclodextrins [10,11,12], crown ether [13,14], antibiotics [15,16], proteins [17], and polysaccharides [18]. A huge number of antibiotics have been used as chiral selectors in CE [19,20], many of which are macrocyclic antibiotics [21,22,23].

Clindamycin, shown in Figure 1, is a semisynthetic lincosamide antibiotic derived from lincomycin through the replacement of the C-7 hydroxyl group with a chlorine atom, accompanied by inversion of configuration at this position. Clindamycin phosphate is the water-soluble phosphate ester prodrug of clindamycin. It is a structurally complex antibiotic containing multiple stereogenic centers and functional groups capable of diverse non-covalent interactions. It has been reported as a chiral selector for basic drugs [24]. Moreover, the use of ionic liquid based on clindamycin phosphate was also reported in the literature [25,26].

The enantioseparation of aromatic amino acids, including tryptophan (Trp), phenylalanine (Phe), and tyrosine (Tyr), has previously been investigated by CE using several classes of chiral selectors [27,28,29,30]. Wan and Blomberg reviewed the principal CE strategies used for the chiral separation of amino acids and peptides, demonstrating that enantioselectivity is strongly influenced by the chemical structure and charge of the selector, the composition and pH of the background electrolyte, and the nature of the interactions formed with the amino acid enantiomers [28]. La et al. compared neutral and charged cyclodextrin selector modes for the enantioseparation of aromatic amino acids, showing that cyclodextrin charge and inclusion complexation play important roles in controlling enantiomer migration and resolution [27]. Suliman et al. subsequently investigated α- and β-cyclodextrins in combination with 18-crown-6 for the separation of aromatic amino acid enantiomers and used molecular modeling to clarify the contribution of host–guest inclusion and complementary intermolecular interactions to chiral recognition [29]. More recently, sulfated β-cyclodextrin was successfully employed as a single chiral selector for the CE enantioseparation of aromatic amino acids, with computational calculations supporting differences in the stability and binding modes of the diastereomeric selector–analyte complexes [30].

Despite these advances, most reported methods for Trp, Phe, and Tyr have relied predominantly on cyclodextrin-based selectors, either alone or in combination with crown ethers. The potential of structurally complex antibiotic molecules as alternative chiral selectors for these amino acids remains comparatively underexplored.

In this study, clindamycin phosphate was used as a chiral selector for the capillary electrophoretic separation of three aromatic amino acids: Trp, Phe, and Tyr. To clarify the underlying chiral-recognition mechanism, CE was combined with NMR spectroscopy and molecular modeling, enabling the observed enantioseparation behavior to be directly linked to selector–analyte interactions at the molecular level. Molecular docking simulations were also conducted to examine the binding modes and relative affinities of the individual amino acid enantiomers. The results revealed the contributions of hydrogen bonding, hydrophobic contacts, and electrostatic interactions to the formation and stabilization of the diastereomeric complexes. These computational findings help explain the differences in electrophoretic mobility and separation efficiency observed experimentally. Overall, this integrated experimental and computational investigation provides valuable insight into the chiral-recognition properties of clindamycin phosphate and demonstrates the potential of antibiotic-based selectors for the enantioselective analysis of amino acids.

## 2. Experimental

### 2.1. Instrumentation

The chiral separation was carried out on a CE instrument from Agilent Technologies, (Waldbronn, Germany), model G7100A, connected to a photo diode array detector and with Agilent Chemstation Software (C.01.01). An uncoated fused-silica capillary of 50 µm i.d × 56 cm (detection length, 8.5 cm from the outlet end of the capillary) from Agilent Technologies was used. A lab pH meter inoLab^®^ pH 7110 was utilized for measuring the pH of the buffer (Xylem analytic Inc., Waldheim, Germany).

### 2.2. Chemicals and Reagents

DL-Tryptophan, DL-Phenylalanine, DL-Tyrosine, L-Tryptophan, L-Phenylalanine, L-Tyrosine and clindamycin phosphate were obtained from Sigma-Aldrich (Steinheim, Germany). All other chemicals were of analytical reagent grade and were used without further purification, acquired from Sigma-Aldrich (St Louis, MO, USA). Sodium phosphate dibasic heptahydrate and orthophosphoric acid (85%) were obtained from Sigma-Aldric (Seelz, Germany). All solutions were prepared utilizing Milli-Q water (Millipore, Bedford, MA, USA).

### 2.3. CE Chiral Separation Conditions

The chiral analysis was performed using the following conditions: temperature of 20 ± 0.1 °C, applied voltage of +19 kV, injection time of 5 s hydrostatically at 0.5 psi and detector wavelength of 214 nm. A capillary was conditioned by rinsing with 1 M sodium hydroxide for 20 min, followed by 0.1 M sodium hydroxide for 8 min and water and background electrolyte (BGE), each for 10 min. The BGE comprised 40 mM borate adjusted to the desired pH with sodium hydroxide. All standards, samples and BGE solutions were filtrated using a syringe filter with a pore size of 0.20 µm, and a diameter of 13 mm (Macherey-Nagel GmBh & Co. KG, Duren, Germany). Prior to switching each BGE to a new buffer solution, the capillary was rinsed with 1 M sodium hydroxide, Milli-Q water and an appropriate BGE for 15, 20, and 30 min, respectively. Each day, the capillary was equilibrated with 0.1 M sodium hydroxide, Milli-Q water and the working buffer solution for 5, 15 and 20 min, respectively. Between the runs the capillary was washed with 0.1 M sodium hydroxide and Milli-Q water each for 2 min, and then with the BGE for 3 min.

### 2.4. Molecular Docking

Molecular docking simulations were carried out to explore the chiral recognition mechanism of clindamycin phosphate toward aromatic amino acids. The three-dimensional structures of clindamycin phosphate and of the D- and L-enantiomers of tryptophan, tyrosine, and phenylalanine were built using the LigPrep module of Schrödinger Maestro (Schrödinger, LLC, New York, NY, USA). Appropriate ionization states of the amino acids and clindamycin phosphate at pH 7.6 and 7.3 were generated, and all structures were energy-minimized using the OPLS4 force field (all amino acids adopted zwitterionic forms). A grid box encompassing the entire clindamycin phosphate molecule was defined to guide the docking process, and each amino acid enantiomer was docked against clindamycin phosphate using the Glide docking algorithm in standard precision (SP) mode. For each enantiomer, the top-scoring pose was retained, and its binding free energy (ΔG_bind) was refined with the Molecular Mechanics–Generalized Born Surface Area (MM-GBSA) method in Prime, using the VSGB 2.0 implicit solvation model and the OPLS4 force field. This approach estimates the binding free energy (ΔG_bind) by combining the molecular mechanics energy (ΔE_MM), the solvation free energy (ΔG_solv), and the surface area contribution (ΔG_SA) for the complex, the free receptor, and the free ligand. Non-covalent interactions in each complex—hydrogen bonding, hydrophobic contact, and electrostatic (charged-group) interactions—were identified from the docked poses, and characteristic interatomic distances were measured. In particular, distances between the tertiary amine and the hydroxyl-bearing sugar region of clindamycin phosphate and the carboxylate and aromatic groups of the amino acids were measured to assess the stability of each complex. Three-dimensional interaction diagrams generated by Maestro were used to visualize the molecular interactions between clindamycin phosphate and each amino acid. The results are summarized in Table 2, which lists the docking scores, MM-GBSA binding free energies, and the types of interactions (hydrogen bonds, hydrophobic contacts, and charge interactions) for each of the six amino acid enantiomers.

### 2.5. NMR Measurements

^1^H NMR spectra were obtained at 25 °C with a JEOL 600 MHz spectrometer operating at 600.00 MHz (JEOL Ltd., Tokyo, Japan), using the HOD signal at δ 4.65 ppm as an internal standard. The sample solutions were prepared by dissolving appropriate amounts of analytes (3.0 mg DL-AAs or L-AAs and/or 5.68 mg clindamycin) in 0.6 mL of D_2_O. Sodium deuteroxide was used to adjust the pH to 7.3 or 7.6. The spectra were obtained under the following conditions: acquisition time, 400 ms; relaxation delay, 3.0 s; and number of scans, 32.

### 2.6. Calculation of Selectivity Factor and Resolution

The apparent selectivity factor (*α*) was calculated from the migration times of the two enantiomers using Equation (1)

*α* = *t*_2_/*t*_1_ (1)

where *t*_1_ and *t*_2_ are the migration times of the first- and second-migrating enantiomers, respectively, with *t*_2_ > *t*_1_. An *α* value greater than 1 indicates differential migration and, therefore, chiral discrimination, whereas *α* = 1 indicates the absence of enantioselectivity.

The enantiomeric resolution (*Rs*) was calculated using Equation (2)

*Rs* = 2(*t*_2_ − *t*_1_)/(*w*_1_ + *w*_2_) (2)

where *w*_1_ and *w*_2_ are the baseline peak widths of the first- and second-migrating enantiomers, respectively. An *Rs* value of approximately 1.5 or higher was considered indicative of baseline separation.

## 3. Results and Discussion

### 3.1. Optimization CE Separation Conditions

#### 3.1.1. Effect of the pH of the BGE

The pH of the BGE has an impact on the charge of the chiral selector and analyte and therefore on electrophoretic mobilities. The pH of BGE was investigated in the range of 6.7 to 8.0 containing clindamycin phosphate at a concentration of 60 mM. The dependence of the enantioseparation on the pH of BGE is shown Table 1. The optimum pH for the chiral separation of the three amino acids under investigation is 7.3 for Phe, and 7.6 for Trp and Tyr. 

#### 3.1.2. Influence of Clindamycin Phosphate Concentration

**Figure 2:**
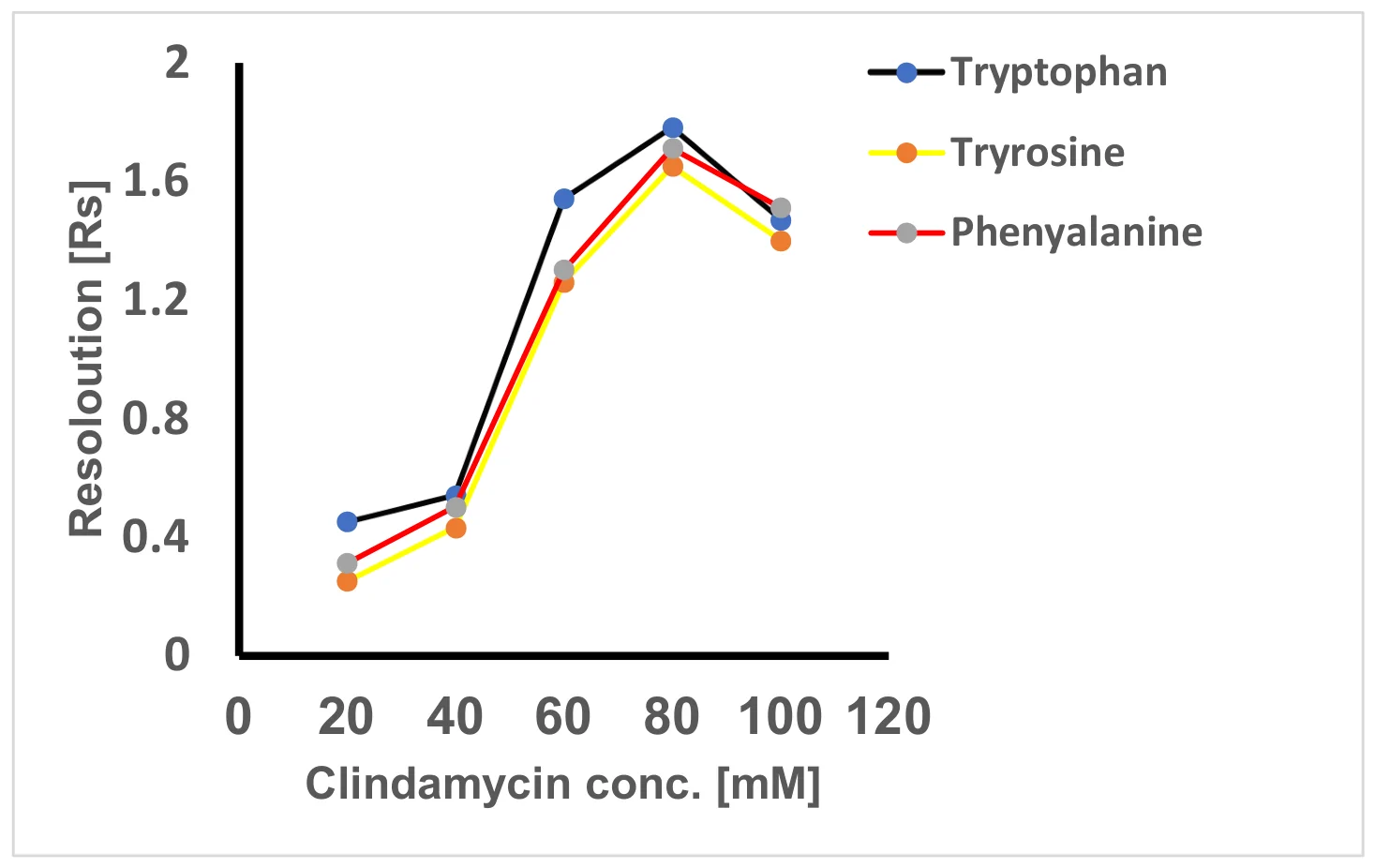
Effect of clindamycin phosphate concentration on the resolution of the amino acids (AAs) (buffer concentration 40 mM, pH 7.3 for phenylalanine and 7.6 for tryptophan and tyrosine, temperature 24 °C, and applied voltage 22 kV).

To identify the optimum chiral-selector concentration for resolving all analytes, the concentration of the clindamycin phosphate was examined in the range of 20–90, mM) at pH 7.3 for Phe and pH 7.6 for Trp and Tyr. The resolution (*Rs*) generally improved as the clindamycin phosphate concentration increased from 20 to 80 mM as shown in Figure 2. However, further increasing the concentration to 100 mM reduced the resolution of all the three amino acids. This decline may be attributed to greater peak broadening and saturation of the complexation equilibrium between the analytes and the chiral selector at higher clindamycin phosphate concentrations. Based on these findings, clindamycin phosphate concentrations of 80 mM provided the most effective enantioseparation of the studied amino acids.

#### 3.1.3. Influence of Organic Solvent Concentration

The addition of an organic modifier to the background electrolyte (BGE) can markedly affect chiral recognition, although its influence depends on both the antibiotic selector and the nature of the analyte. Organic modifiers may alter several physicochemical and electrophoretic properties, including BGE viscosity, the mobilities of the analytes and chiral selector, inclusion complexation—or competition between the solutes and modifier—the solubility of the enantiomers, and electroosmotic flow (EOF) through changes in the dielectric constant and the zeta potential of the capillary wall [31]. Moreover, organic modifiers may strengthen the complexation interactions responsible for enantiomeric discrimination when antibiotics are employed as chiral selectors [15].

The addition of organic solvents, such as protic methanol or aprotic acetonitrile (ACN), to an antibiotic-containing background electrolyte (BGE) has been reported to play an important role in enantiomeric resolution (*Rs*) [5]. Accordingly, the influence of methanol concentration (10–40%, *v*/*v*) on the chiral separation of the investigated enantiomers was systematically evaluated. Increasing the methanol content generally prolonged analyte migration times, most likely because of changes in BGE viscosity, electroosmotic flow (EOF), and analyte interactions with the antibiotic chiral selector. The highest resolution values were achieved at methanol concentrations of 30% (*v*/*v*).

#### 3.1.4. Influence of Operating Parameters

The influence of capillary temperature on enantiomer migration and resolution was investigated over the range of 16–28 °C using 80 mM clindamycin phosphate at the optimum pH, and a methanol concentration of 30% was established for each amino acid. Increasing the temperature generally shortened migration times and reduced enantiomeric resolution (*Rs*). This behavior can be attributed to the lower viscosity of the BGE at elevated temperatures, which alters the electrophoretic mobilities of both the analytes and the chiral selector. Under these conditions, the enantiomers and electroosmotic flow (EOF) migrated toward the cathode, whereas clindamycin phosphate migrated toward the anode. Therefore, the observed reduction in migration times with increasing temperature suggests that the interactions between the enantiomers and clindamycin phosphate became progressively weaker. The best resolution was obtained at 20 °C.

The applied voltage influences three key aspects of capillary electrophoresis: separation efficiency, enantiomeric resolution (*Rs*), and migration time. Increasing the voltage generally improves electrophoretic efficiency and shortens the analysis time; however, it can also intensify Joule heating, resulting in band broadening and poor migration-time reproducibility. Therefore, a relatively high voltage is desirable, provided that excessive Joule heating is avoided. The influence of applied voltage was examined over the range of 16–25 kV. As the voltage increased, the migration times of all enantiomers decreased because of the greater electrophoretic velocities of the analytes and electroosmotic flow (EOF). In contrast, enantiomeric resolution changed only slightly. The best resolution with good migration time was an obtained voltage of 19 kV.

**Figure 3:**
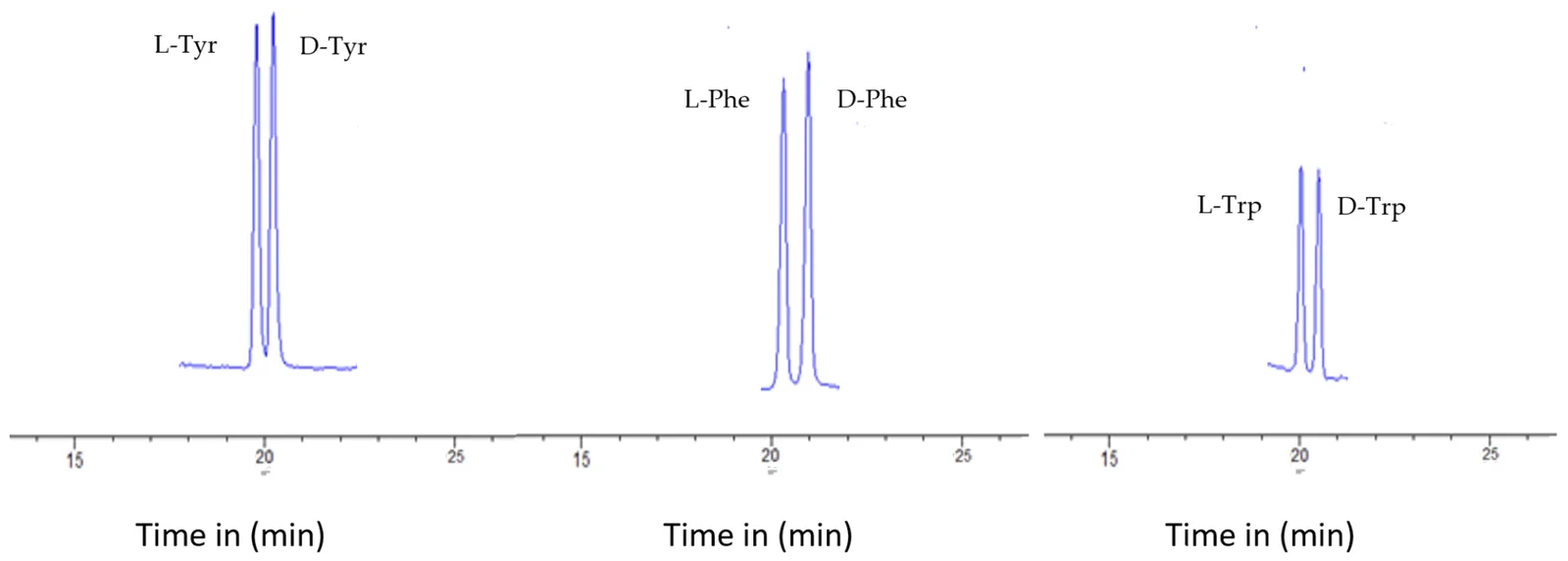
Typical electropherograms for the three amino acids obtained at the optimum conditions, as described in the text.

Based on these findings, the optimum CE conditions consisted of a 40 mM borate buffer (pH 7.3 and 7.6) containing 80 mM clindamycin, with 30% methanol, an applied voltage of 19 kV, and a capillary temperature of 20 °C. The electropherogram obtained for the CHL enantiomers under the optimized conditions is presented in Figure 3. The migration order was identified by spiking L-enantiomers.

### 3.2. Molecular Docking Results

Table 2 summarizes the docking scores and MM-GBSA binding free energies for the six amino acid enantiomers docked against clindamycin. Across all three amino acid pairs, binding affinity followed the order Trp > Phe > Tyr. D- and L-Tyr showed the most favorable docking scores (−4.195 and −3.774 kcal/mol, respectively) and MM-GBSA energies (−24.210 and −22.370 kcal/mol). Phenylalanine gave intermediate values (−3.542/−3.450 kcal/mol; −20.880/−19.850 kcal/mol), while tyrosine showed the weakest binding (−3.108/−3.063 kcal/mol; −18.861/−19.507 kcal/mol). This trend is consistent with the larger, more hydrophobic indole side chain of tryptophan making more extensive contact with clindamycin phosphate than the phenyl ring of phenylalanine or the polar phenolic ring of Tyr.

Because enantioseparation is governed by the difference in binding affinity between the two diastereomeric selector–analyte complexes rather than by the absolute affinity of either enantiomer, the differences in docking score and MM-GBSA binding free energy between the D- and L-enantiomers (ΔΔG = ΔG_D − ΔG_L) are also reported in Table 2. The computed ΔΔG values follow the order Trp (−0.421 kcal/mol docking; −1.840 kcal/mol MM-GBSA) > Phe (−0.092; −1.030) > Tyr (−0.045; +0.646). This trend correlates well with the experimental enantioseparation: Trp, which shows the largest computed enantioselectivity, also exhibited the highest resolution (*Rs* ≈ 1.67) in CE, whereas Tyr, with the smallest and even sign-inconsistent ΔΔG, showed the lowest and most dynamic enantiodiscrimination.

**Figure 4:**
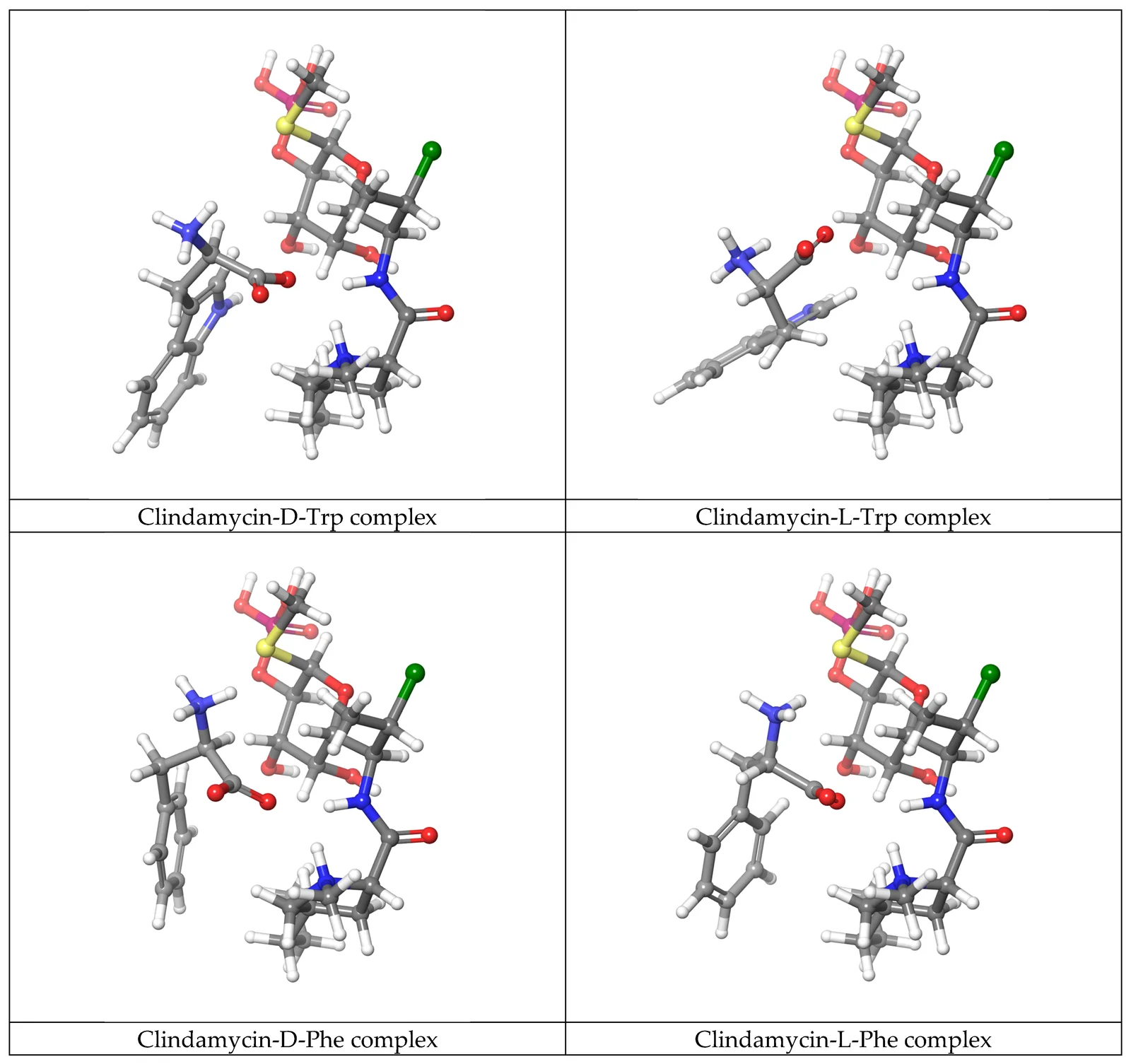

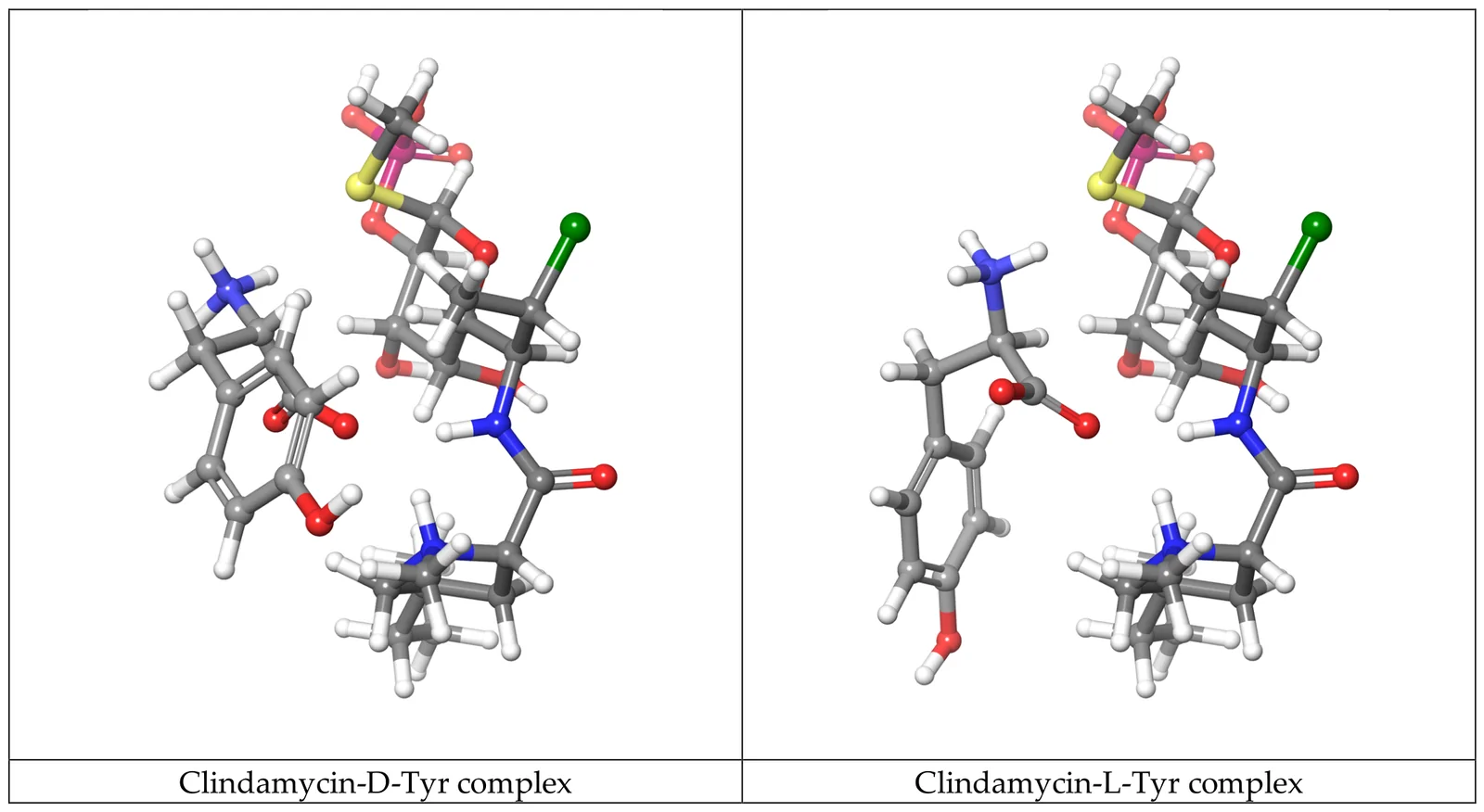
Docking results showing the interacting functional groups and interaction distances (hydrogen bonding, hydrophobic binding, and charge binding) for the clindamycin phosphate (Cly)–amino acid (AA) enantiomer complexes.

The interaction maps in Figure 4 show that the superior affinity of the Trp complexes arises from the simultaneous contribution of several non-covalent interactions. This discrimination correlates with the number and quality of contacts each enantiomer forms. D-Tryptophan engages the full set of interactions captured in the model: a short-range charge interaction between the tertiary amine of clindamycin phosphate and the carboxylate of the amino acid (2.05 Å), a close hydrogen bond between the hydroxyl-bearing sugar region of clindamycin phosphate and the pyrrole-NH of the indole ring (1.97 Å), and a hydrophobic contact (4.01 Å). This gives it the most complete three-point contact network of the six species and, accordingly, the strongest overall binding. L-Trp retains the hydrogen bond (1.82 Å) and a comparable hydrophobic contact (4.35 Å), but its charge interaction is considerably longer (3.47 Å versus 2.05 Å), consistent with weaker electrostatic pairing and a lower binding energy relative to the D-form.

Phe, by contrast, forms no measurable hydrogen bond in either enantiomer, relying only on hydrophobic (3.26/3.29 Å) and charge (3.47/3.67 Å) contacts. The absence of a hydrogen-bonding contact likely accounts for its intermediate binding affinity relative to tryptophan. Tyr shows the weakest overall binding of the series. Notably, its hydrogen bond and hydrophobic distances (6.04 and 6.02 Å for the D-form; 7.03 and 3.29 Å for the L-form) are considerably longer than the corresponding contacts in Trp and Phe. For D- and L-Tyr, values above ~6 Å likely indicate a functional group positioned outside effective interaction range rather than a genuine contact, which is effectively equivalent to the absent contacts seen for phenylalanine. This loss of engagement at one or more contact points is consistent with tyrosine’s position at the bottom of the binding-affinity ranking.

These results suggest that clindamycin’s ability to discriminate D- from L-enantiomers scales with the number of simultaneous contact points each enantiomer can form—most complete for tryptophan, partial for Phe, and weakest for Tyr—providing a molecular rationale for the differential chiral recognition observed in the capillary electrophoresis separation.

Overall, the computational results indicate the following binding affinity trend:

D-Trp > L-Trp > D-Phe > L-Phe > D-Tyr ≈ L-Tyr.

This trend reflects the increasing contribution of aromatic surface area and cooperative hydrogen-bonding interactions, highlighting the role of the tryptophan indole ring in stabilizing clindamycin phosphate through multiple complementary non-covalent interactions. The agreement between Glide docking scores and MM-GBSA binding free energies further supports the reliability of the predicted interaction pattern.

### 3.3. Integrated NMR and Molecular Docking Analysis

This section integrates the semiquantitative 1D ^1^H NMR chemical-shift perturbation (CSP) analysis with molecular docking (Schrödinger Glide SP and MM-GBSA) to characterize the chiral recognition of three aromatic amino acids (phenylalanine, tryptophan, and tyrosine; L- and DL-forms) by clindamycin phosphate in D_2_O. Docking predicts atom-level binding poses and interaction distances, while NMR experimentally identifies which molecular regions are perturbed upon complex formation. Several points should be noted: (1) no 2D NMR (NOESY/ROESY, DOSY) or titration data are available, so proton-by-proton assignments rely on literature values [32,33,34,35,36,37,38,39,40], and association constants cannot be determined; (2) the DL-racemic spectra contain both enantiomers, so perturbations unique to DL versus L suggest D-enantiomer contributions; (3) the uniform shift in the propyl terminal CH_3_ (~0.04 ppm across all mixtures) likely reflects non-specific environmental effects rather than direct binding contacts; and (4) line broadening in all mixtures is consistent with intermediate chemical exchange on the NMR timescale. Detailed information on the ^1^H NMR signal envelopes of each amino acid and of clindamycin phosphate in D_2_O can be found in Table S1 (clindamycin phosphate) and Table S2 (amino acids) in the Supplementary Materials. Accordingly, the CSP and line-broadening observations are interpreted as evidence for changes in the local chemical environment and exchange behavior of clindamycin phosphate upon amino acid association, rather than as direct proof of individual intermolecular contacts or a unique docking pose.

Table 3 summarizes the clindamycin phosphate regions perturbed upon binding, ranked by confidence. The pyrrolidine CH_2_ envelope (~2.0 ppm) is the most consistently perturbed signal across all six mixtures, exhibiting strong downfield shifts of +0.043 to +0.071 ppm (Perturbation tiers: STRONG ≥ 0.05 ppm; MODERATE 0.02–0.05 ppm; WEAK 0.01–0.02 ppm; NEGLIGIBLE < 0.01 ppm). This region corresponds to the protonated tertiary amine, which docking (Table 2) predicts forms an electrostatic ion pair with the amino acid carboxylate (COO^−^) at distances of 2.05–3.67 Å. The anomeric/thiosugar region (~5.38 ppm) shows moderate-to-strong downfield shifts (+0.04 to +0.05 ppm) in five of six mixtures, with peak splitting observed for L-Trp, indicating exchange between free and bound states. The hydroxyl-bearing sugar methines (H-3/H-4, ~4.1–4.5 ppm) are strongly perturbed, particularly in DL-Phe (+0.068), DL-Trp (+0.075), and L-Tyr (+0.082), supporting involvement of the polar sugar face in binding; docking predicts a direct sugar-OH → indole-NH hydrogen bond specifically for tryptophan. The methylthio S–CH_3_ (~2.75 ppm) shows moderate downfield shifts (+0.043 to +0.052) in Phe and Trp mixtures, with peak splitting in DL mixtures indicating free/bound exchange. The C-8 methyl (~1.26 ppm) displays a consistent upfield shift (−0.036 to −0.063 ppm) in five of six mixtures, a chemical-shift change consistent with altered magnetic shielding in an aromatic environment; the corresponding proximity to an aromatic ring is predicted by the docking models.

The docking and NMR results provide complementary, but not equivalent, information on the molecular recognition process. The NMR data experimentally demonstrate changes in the chemical environments and line shapes of several clindamycin phosphate regions upon addition of the amino acids, whereas the docking calculations provide structural models that propose possible intermolecular contacts and their approximate geometries. On this basis, the combined data are consistent with a multipoint recognition model involving electrostatic, hydrophobic, and hydrogen-bonding contributions; however, the individual docking contacts should be regarded as model-derived interpretations rather than as direct experimental observations.

(1) Charge interaction: The pyrrolidine tertiary amine region exhibits reproducible chemical-shift perturbations across the amino acid mixtures. The docking models predict an electrostatic interaction between this protonated region and the amino acid carboxylate, with calculated distances of 2.05–3.67 Å. Thus, the NMR perturbation is consistent with involvement of the cationic region, while the specific ion-pair geometry is inferred from docking.

(2) Aromatic/hydrophobic interaction: The C-8 methyl region shows upfield chemical-shift changes in several amino acid mixtures. These changes are consistent with an altered magnetic environment associated with interaction with an aromatic amino acid moiety. The docking models provide a possible structural explanation by placing the C-8 methyl/alkyl region near the aromatic rings of Trp and Phe. The NMR data therefore support involvement of this region but do not independently establish the precise CH_3_-aromatic contact geometry.

(3) Hydrogen-bonding contribution: The hydroxyl-bearing sugar methine region exhibits measurable perturbations, particularly in Trp-containing samples. The docking calculations predict hydrogen-bonding interactions involving sugar hydroxyl groups for selected complexes. The NMR observations are therefore compatible with involvement of the polar sugar region, while the specific hydrogen-bond geometry remains a docking-derived hypothesis.

(4) Thiosugar/methylthio region: Perturbations and line-shape changes are observed around the methylthio region, particularly in Phe- and Trp-containing mixtures. Docking predicts contacts between this region and functional groups of the amino acids in selected poses. These NMR observations indicate changes in the local environment and exchange behavior of the methylthio region but do not by themselves establish a unique sulfur-mediated intermolecular contact.

Overall, the combined CE, NMR, and docking results support a complementary mechanistic model in which multiple molecular regions of clindamycin phosphate participate in amino acid recognition. Nevertheless, because the present NMR analysis is based primarily on 1D chemical-shift and line-shape changes without 2D intermolecular NOE/ROE or quantitative titration data, the docking-predicted contacts should be regarded as plausible structural interpretations rather than experimentally confirmed intermolecular contacts.

As shown in Table 2, docking predicts the binding affinity trend D-Trp > L-Trp > D-Phe > L-Phe > D-Tyr ≈ L-Tyr (docking scores −4.195 to −3.063 kcal/mol; MM-GBSA −24.210 to −18.861 kcal/mol). The NMR chiral recognition evidence is summarized in Table 4. For phenylalanine, the racemic mixture produces a 7-fold larger aromatic CSP (+0.090 ppm) compared with the L-enantiomer alone (+0.013 ppm), along with a reversal of Hα/Hβ shift directions between L and DL samples—the clearest experimental evidence of enantiomer-dependent binding geometry. For tryptophan, L-Trp shows stronger indole proton perturbations (+0.053 to +0.056 ppm) and peak splitting, consistent with slower exchange and possibly a longer-lived complex. However, this cannot be directly converted into a D-versus-L affinity ranking because the NMR comparison is L versus DL, not pure L versus pure D. The DL-Trp mixture produces stronger clindamycin phosphate sugar-methine perturbation (+0.114 versus +0.089 ppm for L-Trp), consistent with a D-enantiomer contribution predicted by docking. For tyrosine, docking predicts near-degenerate D/L energies with several contact distances > 6 Å (outside effective range), yet the DL-Tyr mixture shows the most dramatic spectral change: complete loss of aromatic proton signals due to extreme intermediate-exchange broadening, reflecting dynamic heterogeneity rather than strong thermodynamic affinity. Overall, chiral discrimination arises from the stereochemical complementarity of these contacts. When the enantiomer is swapped, some contacts shorten while others lengthen. For tryptophan, D-Trp shortens the charge distance (2.05 versus 3.47 Å for L) and gains the sulfur contact (4.04 Å), while maintaining the hydrogen bond—the most complete contact network predicted by docking. For phenylalanine, D-Phe slightly shortens both the hydrophobic and charge distances in the docking models, while the larger aromatic CSP and Hα/Hβ direction reversal observed for the DL mixture are consistent with an enantiomer-dependent change in the local chemical environment. For tyrosine, docking shows near-degenerate D/L energies, but D-Tyr loses the hydrophobic contact (6.02 Å, outside range) while L-Tyr maintains it (3.29 Å). The extreme broadening in DL-Tyr reflects dynamic heterogeneity from multi-pose binding—a kinetic dimension of chiral recognition that is invisible to static docking but clearly detected by NMR line-shape analysis.

In summary, clindamycin phosphate acts as a chiral selector by presenting a multifunctional binding surface—polar sugar/phosphate, hydrophobic methylthio/alkyl, and cationic pyrrolidine—that engages aromatic amino acids through complementary contacts whose geometry differs between enantiomers. The number, quality, and dynamics of these contacts determine the degree of chiral discrimination: tryptophan achieves the most complete and stable recognition (strongest computed affinity, most complete contact network), phenylalanine shows the clearest experimental evidence of enantiomer-dependent binding geometry (7-fold CSP amplification, Hα/Hβ reversal), and tyrosine reveals the role of binding dynamics in chiral recognition (extreme exchange broadening despite weak docking discrimination). The charge interaction is the most consistently observed NMR perturbation and is also predicted in the docking models across the investigated systems, supporting its possible role as an important anchoring interaction.

## 4. Conclusions

In conclusion, clindamycin phosphate was successfully employed as a chiral selector for the capillary electrophoretic separation of Trp, Tyr, and Phe enantiomers. Effective separation was achieved by optimizing the BGE pH, clindamycin phosphate and methanol concentrations, capillary temperature, and applied voltage; the best conditions were 40 mM borate (pH 7.3 for Phe, 7.6 for Trp and Tyr) containing 80 mM clindamycin phosphate and 30% (*v*/*v*) methanol, at 19 kV and 20 °C. Experimentally (^1^H NMR CSP analysis of free and bound states), complexation of the amino acids by clindamycin phosphate involves the pyrrolidine ammonium region, the hydroxyl-bearing sugar face, the methylthio group and the C-8 methyl group, with exchange broadening indicating intermediate-exchange binding kinetics; for Phe, the 7-fold larger aromatic CSP in the DL versus the L sample, together with the reversal of the Hα/Hβ shift directions, provides direct experimental evidence of enantiomer-dependent binding geometry. Molecular docking and MM-GBSA calculations predicted electrostatic attraction between the clindamycin phosphate tertiary amine and the amino acid carboxylate as the dominant binding anchor, supported by hydrophobic and hydrogen-bonding contacts, a pattern consistent with the enantiomer-dependent ^1^H NMR chemical-shift perturbations experimentally observed for clindamycin phosphate upon complexation. The computational results indicate a clear D-enantiomer preference for tryptophan and, to a lesser extent, phenylalanine, whereas for tyrosine the docking score and MM-GBSA energy disagree in sign and the computed discrimination falls within the uncertainty of the methods; the pronounced NMR line broadening observed for DL-tyrosine instead indicates dynamic, exchange-mediated binding behavior not captured by static docking. We emphasize that the specific intermolecular contacts and distances discussed above are model-derived (Glide SP/MM-GBSA) and have not been independently confirmed by 2D NMR or structural methods; they represent a plausible, NMR-consistent working model of the recognition mechanism rather than a directly proven binding geometry. Overall, this integrated CE-docking–NMR study demonstrates the potential of clindamycin phosphate as an effective CE chiral selector for aromatic amino acids and illustrates how combining these complementary techniques provides mechanistic insight beyond what any single method affords.

The NMR results experimentally demonstrated chemical-shift perturbations and line-broadening behavior in several clindamycin phosphate regions, while molecular docking provided plausible structural models for the corresponding intermolecular interactions. The agreement between these complementary approaches supports a multipoint recognition mechanism involving electrostatic, hydrophobic, and hydrogen-bonding contributions; however, the specific contacts and binding geometries inferred from docking should not be regarded as directly established by the 1D CSP data alone.

## Figures and Tables

**Table 1:** Effect of BGE pH on the selectivity (*α*) and resolution (*Rs*) of aromatic amino acids *.

Chiral AAs	pH									
	6.7		7.0		7.3		7.6		8	
	*α*	*Rs*	*α*	*Rs*	*α*	*Rs*	*α*	*Rs*	*α*	*Rs*
**Phe**	1.010	<1.0	1.03	1.21	1.05	1.48	1.02	1.32	1.01	1.49
**Tyr**	1.004	<1.0	1.01	0.98	1.03	1.47	1.05	1.58	1.03	1.52
**Trp**	1.002	<1.0	1.01	0.92	1.02	1.20	1.05	1.67	1.04	1.61

* Conditions: fused-silica capillary, 50 cm (41.5 cm effective length), 50 mm id; BGE, 40 mM borax buffer with methanol (20% *v*/*v*) containing clindamycin phosphate to a concentration of 60 mM; buffer pH, as shown in Table 1; applied voltage, 18 kV; capillary temperature, 20 °C; detection 214 nm.

**Table 2:** Docking scores, MM-GBSA dG bind energies, interacting functional groups, and length of hydrogen bonding, hydrophobic binding, and charge binding of the clindamycin phosphate (Cly)–amino acid (AA) enantiomer complexes: best values.

Amino Acid	Δ Docking Score (D − L), kcal/mol	SP Docking Score kcal/mol	Δ MM-GBSA ΔG (D − L), kcal/mol	MM-GBSA dG Bind kcal/mol	Hydrogen Bonding				Hydrophobic Binding		Charge Binding	
					Sulfur Atom, Cly.	NH^3+^, AA	Cyclohexane-OH, Cly.	Pyrol-NH Ring, AA	CH^3^, Cly.	Aromatic Ring, AA	Tertiary Amine, Cly.	CO_2_^−^, AA
D-Trp	−0.421	−4.195	−1.840	−24.210	4.04		1.97		4.01		2.05	
L-Trp		−3.774		−22.370	/		1.82		4.35		3.47	
D-Phe	−0.092	−3.542	−1.030	−20.880	/		/		3.26		3.47	
L-Phe		−3.450		−19.850	/		/		3.29		3.67	
D-Tyr	−0.045	−3.108	+0.646	−18.861	6.04		/		6.02		3.47	
L-Tyr		−3.063		−19.507	7.03		/		3.29		3.47	

**Table 3:** Clindamycin phosphate interaction sites ranked by NMR confidence and mapped to docking-predicted contacts.

Clindamycin Phosphate Region (δ, ppm)	Δδ Range (ppm)	Confidence	Docking-Predicted Interaction
Pyrrolidine CH_2_ (~2.0)	+0.043 to +0.071	HIGH	Tertiary amine–COO^−^ charge interaction (2.05–3.67 Å)
Anomeric/thiosugar (~5.38)	+0.04 to +0.05	HIGH	S-CH_3_/thiosugar face; peak splitting in L-Trp
Hydroxyl sugar methines (~4.1–4.5)	+0.02 to +0.08	HIGH	Sugar-OH → amino acid NH H-bond (1.82–1.97 Å for Trp)
S-CH_3_ methylthio (~2.75)	+0.043 to +0.052	MODERATE	S atom → NH^3+^contact; exchange-induced splitting in DL
C-8 methyl (~1.26)	−0.036 to −0.063	MODERATE	CH_3_ → aromatic ring (ring-current shielding; 3.26–4.35 Å)
N-CH_3_ pyrrolidine (~2.19)	+0.004 to +0.047	MODERATE	Tertiary amine region; strongest for L-Tyr
Propyl terminal CH_3_ (~0.71)	+0.034 to +0.047	LOW	Non-specific (uniform across all mixtures)

Δδ values reported as δ(mixture) − δ(free clindamycin). Positive = downfield, negative = upfield.

**Table 4:** Per-amino acid integrated docking–NMR analysis of chiral recognition by clindamycin phosphate.

Amino Acid	Docking Finding	NMR Finding	Chiral Discrimination Evidence
Phe	Intermediate affinity (−3.542/−3.450 kcal/mol); 2-point binding (hydrophobic + charge); no H-bond; D slightly favored	DL aromatic shift +0.090 vs. L +0.013 ppm (7×); Hα/Hβ direction reversal; C-8 methyl upfield (−0.048 to −0.055)	Strongest evidence for enantiomer-dependent chemical-environment changes; Docking predicts different D/L orientations; NMR shows corresponding differences in chemical-shift behavior
Trp	Strongest affinity (−4.195/−3.774 kcal/mol); 3-point + sulfur contact; H-bond 1.82–1.97 Å; charge 2.05 Å (D)	L-Trp: stronger indole CSPs, peak splitting; DL-Trp: stronger sugar-methine perturbation (+0.114)	D > L predicted by docking; L tighter by NMR (peak splitting); DL shows D-specific sugar contacts
Tyr	Weakest/near-degenerate (−3.108/−3.063 kcal/mol); D-Tyr distances >6 Å; MM-GBSA favors L	DL-Tyr: aromatic signals completely lost (extreme broadening); L-Tyr: sugar methines +0.082; Hα reversal	NMR reveals strong dynamic differences despite weak docking discrimination; multi-pose exchange

## Data Availability

The original contributions presented in this study are included in the article/Supplementary Materials. Further inquiries can be directed to the corresponding authors.

## Supplementary Materials

The following supporting information can be downloaded at https://www.mdpi.com/article/10.3390/molecules31203580/s1: Table S1: Clindamycin phosphate ^1^H NMR signal envelopes in D2O, assigned based on literature data ^1,2,3,4^.; Table S2: Amino acid CSPs in clindamycin mixtures. For DL mixtures, δ free refers to the DL-amino acid reference spectrum.
