# Supplementary material for: Clindamycin Phosphate-Mediated Enantioseparation of Amino Acids by Capillary Electrophoresis: Molecular Modeling and NMR Insights into the Chiral Recognition Mechanism
Source: Molecules. 2026 Oct 9;31(20):3580. doi: 10.3390/molecules31203580 (PMC13707754; doi:10.3390/molecules31203580)
Supplement: Supplementary file 1 [file molecules-31-03580-s001.zip › molecules-4583535-supplementary.pdf]

Supplementary materials:

Table S1. Clindamycin phosphate ^1H NMR signal envelopes in D_2O, assigned based on literature data[1,2,3,4].

Signal Envelope	δ range (ppm)	Structural Assignment
Anomeric/thiosugar	5.3–5.5	H-1 (anomeric), adjacent to $S–CH_3$
Phosphate-adjacent sugar	4.4–4.5	H-2 (C-2 bears $O–PO_3H_2$)
Hydroxyl-bearing sugar methines	4.1–4.3	H-3, H-4 (hydroxyl-bearing)
Sugar/amide methines	3.4–3.8	H-5, H-6, H-7, pyrrolidine H-1'
Methylthio	2.7–2.8	$S–CH_3$
Pyrrolidine/N-alkyl	2.0–2.3	$N–CH_3$, pyrrolidine ring CH_2
C-8 methyl	1.2–1.3	C-8 methyl doublet
Propyl chain	0.7–1.1	Propyl CH_2 and terminal CH_3

Table S2. Amino acid CSPs in clindamycin mixtures. For DL mixtures, δ free refers to the DL-amino acid reference spectrum.

Amino acid signal	δ free	Δδ in L-mix	Δδ in DL-mix
Phenylalanine			
Aromatic H	7.19/7.15	+0.013	+0.090
Hα	3.80/3.89	+0.012	-0.043
Hβ	3.02/3.31	+0.036	-0.036
Tryptophan			
Indole H-4	7.55	+0.056	+0.048
Indole H-2	7.35	+0.055	+0.047
Indole H-5/H-6	7.10	+0.036	+0.046
Indole H-7	7.01	+0.053	+0.045
Hα	3.87/3.90	+0.046	+0.016
Hβ	3.21/3.22	+0.028	+0.007
Tyrosine			
Aromatic (ortho)	7.01/7.00	+0.037	SIGNAL LOST
Aromatic (meta)	6.71/6.70	+0.043	SIGNAL LOST
Hα	3.75/3.82	+0.044	-0.061
Hβ	2.94/3.22	-0.069	+0.048
